# Clumping and Viability of Bone Marrow Derived Mesenchymal Stromal Cells under Different Preparation Procedures: A Flow Cytometry-Based In Vitro Study

**DOI:** 10.1155/2016/1764938

**Published:** 2016-02-28

**Authors:** Li-li Cui, Tuure Kinnunen, Johannes Boltze, Johanna Nystedt, Jukka Jolkkonen

**Affiliations:** ^1^Institute of Clinical Medicine-Neurology, University of Eastern Finland, 70210 Kuopio, Finland; ^2^Department of Clinical Microbiology, Institute of Clinical Medicine, University of Eastern Finland, 70210 Kuopio, Finland; ^3^Fraunhofer Institute for Cell Therapy and Immunology, 04103 Leipzig, Germany; ^4^Fraunhofer Research Institution for Marine Biotechnology, 23562 Lübeck, Germany; ^5^Institute for Medical and Marine Biotechnology, University of Lübeck, 23562 Lübeck, Germany; ^6^Finnish Red Cross Blood Service, Advanced Cell Therapy Centre, 00310 Helsinki, Finland; ^7^Neurocenter, Neurology, University Hospital of Kuopio, 70210 Kuopio, Finland

## Abstract

Complications of microocclusions have been reported after intra-arterial delivery of mesenchymal stromal cells. Hence, quantification and efficient limitation of cell clumps in suspension before transplantation is important to reduce the risk. We used a flow cytometry-based pulse-width assay to assess the effects of different cell suspension concentrations (0.2–2.0 × 10^6^/mL), storage solutions (complete growth medium, Dulbecco's phosphate-buffered saline, and normal saline), storage time in suspension (0–9 h), and freeze-thawing procedure on the clumping of rat bone marrow derived mesenchymal stromal cells (BMMSCs) and also evaluated cell viability at the same time. Surprisingly, increasing the cell concentration did not result in more cell clumps in vitro. Freshly harvested (fresh) cells in normal saline had significantly fewer cell clumps and also displayed high viability (>90%). A time-dependent reduction in viability was observed for cells in all three storage solutions, without any significant change in the clumping tendency except for cells in medium. Fresh cells were more viable than their frozen-thawed counterparts, and fresh cells in normal saline had fewer cell clumps. In conclusion, cell clumping and viability could be affected by different cell preparation procedures, and quantification of cell clumping can be conducted using the flow cytometry-based pulse-width assay before intra-arterial cell delivery.

## 1. Introduction

Mesenchymal stromal cells (MSCs) can be isolated from various tissues such as bone marrow, umbilical cord blood, and adipose tissue. MSCs are promising candidates for cell therapy because of their multipotency, immunomodulatory effects, easy accessibility, lack of immunogenicity as well as their ethical advantages. Promising positive effects of MSCs administration have been obtained in experimental studies on stroke treatment [1–3] and some early phase clinical trials are currently in progress [4].

Intravascular MSC delivery has been most commonly used in both preclinical and clinical studies with the least invasiveness. However, after intravenous infusion, most cells have been found to be trapped in the internal organs [5], leading to a potential risk of pulmonary embolism [6]. Intra-arterial infusion can increase the cell homing to the ischemic hemisphere since this circumvents the pulmonary circulation [7, 8], but this route carries also a higher risk of complications such as microocclusions [9–12]. For example, in our previous study using allogeneic bone marrow derived mesenchymal stromal cells (BMMSCs), a dose-dependent cerebral embolism was evoked after intra-arterial cell delivery into rats [12]. The relatively large size of MSCs is one important reason for the vascular embolism after cell therapy [11, 13]. Another possible reason is that cell clumps exist in suspension already prior to transplantation. To reduce the potential risk of embolism while maintaining efficacy, it is important to quantify cell clumping and limit the number of large clumps, but so far few studies have addressed this issue.

The flow cytometry-based pulse-width assay has been introduced as a rapid method with a high level of accuracy and sensitivity for quantifying cell clumps [14]. In addition, cell viability, an important in vitro predictor of the efficacy of cell therapy [15], can also be easily evaluated by flow cytometry. During the cell preparation procedure, many variables from ex vivo expansion until delivery might affect the tendency towards cell clumping as well as cell viability. It is very important that, before transplantation, one can be sure that there are limited cell clumps in the cell suspension which has maintained good cell viability. Therefore, we applied the flow cytometry-based assay to assess the effects of different cell suspension concentrations (0.2–2.0 × 10^6^/mL), different storage solutions (complete growth medium, Dulbecco's phosphate-buffered saline and normal saline), storage time in suspension (0–9 h), and freeze-thawing procedure on cell clumping as well as cell viability.

## 2. Materials and Methods

### 2.1. Cell Culture and Characterization of Bone Marrow Derived Mesenchymal Stromal Cells

Oricell*™* male Wistar rat BMMSCs (Cyagen Bioscience Inc., Cat. No. RAWMX-01001) were used in order to be consistent with our previous work [12]. According to the manufacturer's instructions, the cells were cultured in OriCell MSC growth medium supplemented with 10% fetal bovine serum (FBS), 1% glutamine, and 1% penicillin-streptomycin (all reagents are from Cyagen Biosciences Inc., Cat. No. GUXMX-90011). The medium was changed twice every week. The cells were passaged after reaching 80–90% confluency and subcultured at a cell density of 6000 cells/cm^2^. Rat BMMSCs at passage 5 were cryopreserved in the protein-free Oricell*™* NCR cryopreservation medium (Cyagen Biosciences Inc., Cat. No. NCPF-10001). The cells were characterized as previously described [16].

### 2.2. Preparation of Cell Samples for Analysis

Before measurement, cultured rat BMMSCs at passage 5 were harvested using 0.05% trypsin-EDTA (Life Technologies, Cat. No. 25300-054); cryopreserved rat cells were thawed in a water bath at 37°C before being decanted into the complete medium (OriCell MSC growth medium supplemented with 10% FBS, 1% glutamine, and 1% penicillin-streptomycin). After centrifugation at 1000 g for 5 min, freshly harvested (fresh) or frozen-thawed (thawed) cells were resuspended in Dulbecco's phosphate-buffered saline (DPBS) without calcium or magnesium, normal saline (NS; 0.9% NaCl), or complete medium. To analyze the effect of cell concentration on cell clumping and viability in the MSC suspension before transplantation, fresh cells were resuspended in DPBS at a concentration of 0.2 × 10^6^/mL, 0.5 × 10^6^/mL, 1.0 × 10^6^/mL, and 2.0 × 10^6^/mL, respectively. The effect of storage time was assessed by maintaining the cells at 37°C for 3 h, 6 h, and 9 h at a concentration of 0.2 × 10^6^/mL before measurement.

### 2.3. Detection of Cell Clumping Using the Flow Cytometry-Based Pulse-Width Assay

**Figure 1:**
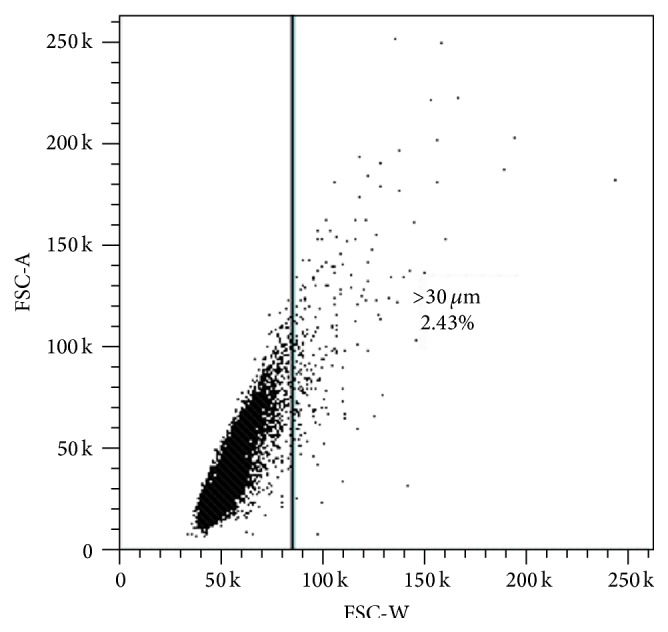
Gating of cell clumps or large cells >30 *µ*m. After the exclusion of debris, FSC-W was plotted against FSC-A and calibrated using standardized polystyrene microspheres.

The pulse width is based on the laser beam height, particle velocity, and particle diameter. Because of the constant sheath pressure which ensures a stable particle velocity, and the constant beam height within the instrument setup, the pulse width can be directly correlated with the diameter of the particle. After calibration of the FSC-W axis by standardized polystyrene microspheres (Polysciences Inc., Cat. No. 64165-15), clumps or large cells >30 *µ*m were gated as suggested by Hickerson et al. [14]. FSC-W was plotted against FSC-A by adjusting the photomultiplier tube voltage and area scaling factor (Figure 1).

### 2.4. Detection of Cell Viability, Apoptosis, and Death by Flow Cytometry

**Figure 2:**
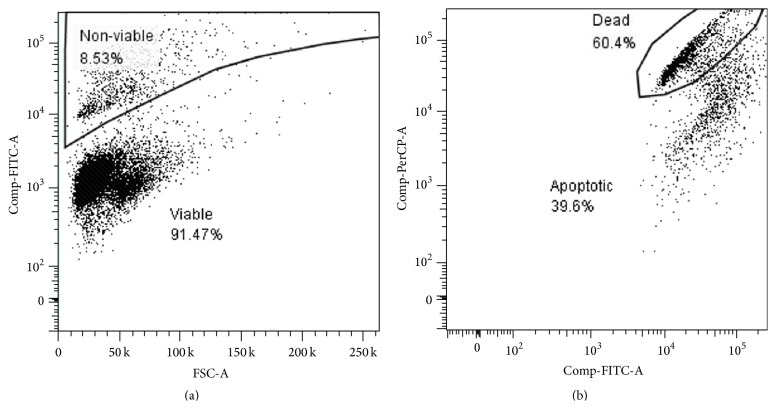
Gating of viable, dead, and apoptotic MSCs. (a) After excluding debris, the CellEvent*™* caspase-3/7 FITC positive cells were gated as nonviable, and the rest as viable cells. (b) Nonviable cells were then gated as dead (SYTOX®AADvanced*™* positive) and apoptotic cells (the rest).

Cell apoptosis and death were assessed with a CellEvent*™* Caspase-3/7 Green Flow Cytometry Assay Kit (ThermoFisher Scientific, Cat. No. C10427) according to the manufacturer's instruction. Briefly, CellEvent*™* Caspase-3/7 Green Detection Reagent was added to the cell suspension and then incubated in the dark at 37°C for 30 minutes. After the final 5 minutes of staining, SYTOX®AADvanced*™* dead cell stain solution in dimethylsulfoxide was added to the suspension. The samples were run on a FACS Canto II flow cytometer (BD Biosciences, USA) using FACSDiva software. The data was analyzed using Flowjo 7.6.2 (Figure 2). Trypan blue staining was also used to determine cell numbers and cell viability in a Countess*™* automated cell counter (Invitrogen, Cat. No. C10281).

### 2.5. Statistical Analysis

Statistical analyses were conducted using the IBM SPSS 21.0 software. Kruskal-Wallis test followed by multiple comparisons was used to compare groups with different cell concentrations or in different storage solutions. Wilcoxon signed-rank test was used to compare fresh and thawed cells. Spearman correlation was used to evaluate the relationship between the two variables. *P* < 0.05 was considered as statistically significant. Results are presented as mean ± standard deviation (SD).

## 3. Results

### 3.1. Effect of Cell Concentration on Cell Clumping and Viability before Transplantation

**Figure 3:**
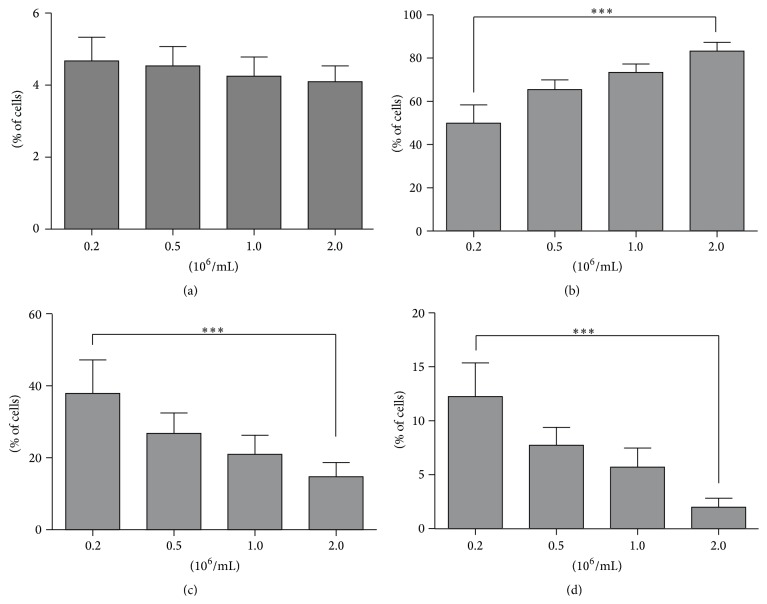
Effect of cell concentration on cell clumping and viability before transplantation. Fresh passage 5 rat BMMSCs were resuspended in 1 mL DPBS with escalating concentrations of 0.2, 0.5, 1.0, and 2.0 × 10^6^/mL, respectively. (a) Cell clumps or large cells >30 *µ*m; (b) viable cells; (c) dead cells; (d) apoptotic cells. ^*∗∗∗*^ *P* < 0.001.

No increase in the extent of cell clumping was observed with escalating concentrations of fresh rat BMMSCs in DPBS (*P* > 0.05). The viability (percentage of viable cells) was significantly higher (*P* < 0.001), while the corresponding numbers of dead and apoptotic cells were significantly lower (*P* < 0.001) in the 2 × 10^6^/mL group than in the 0.2 × 10^6^/mL group (Figure 3).

No significant correlation was found between the percentage of cell clumps and cell concentration (*r* = −0.321, *P* > 0.05). In contrast, the percentage of viable cells was positively correlated (*r* = 0.941, *P* < 0.001), whereas the corresponding values of dead (*r* = −0.831, *P* < 0.001) and apoptotic cells (*r* = −0.913, *P* < 0.001) were negatively correlated with escalating cell concentrations.

### 3.2. Effects of Storage Solution and Storage Time on Cell Clumping and Viability

**Figure 4:**
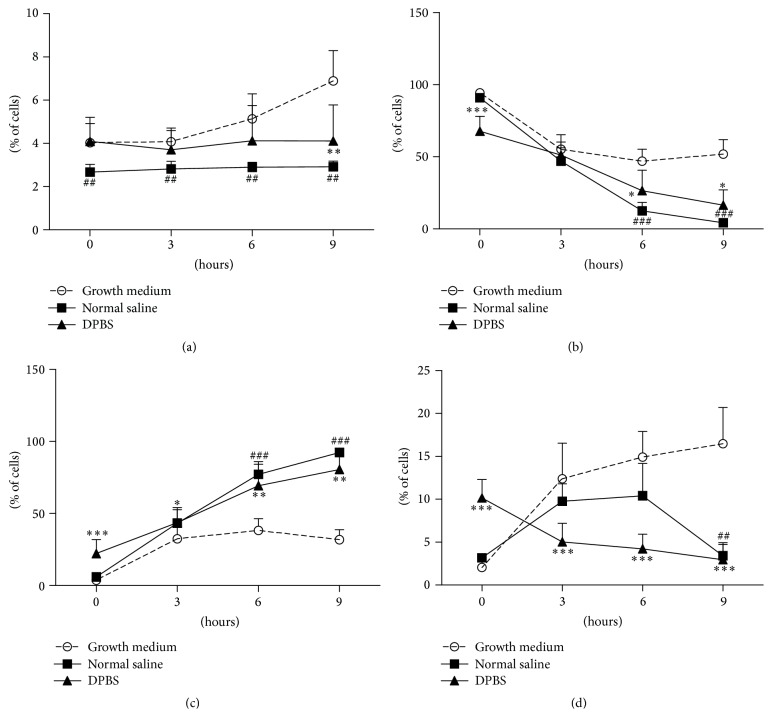
Effects of storage solution and storage time on cell clumping and viability before transplantation. Fresh passage 5 rat BMMSCs were resuspended in complete growth medium, normal saline, and DPBS and stored at 37°C for 3 h, 6 h, and 9 h before analysis. (a) Cell clumps or large cells >30 *µ*m; (b) viable cells; (c) dead cells; (d) apoptotic cells. ^##^ *P* < 0.01 normal saline versus growth medium. ^###^ *P* < 0.001 normal saline versus growth medium. ^*∗*^ *P* < 0.05 DPBS versus growth medium; ^*∗∗*^ *P* < 0.01 DPBS versus growth medium; ^*∗∗∗*^ *P* < 0.001 DPBS versus growth medium.

After harvesting from cell culture (0 h), there were significantly fewer cell clumps preserved in NS (*P* < 0.01 compared to that of cells in DPBS or medium), with viability (>90%), mortality, and apoptosis rates being comparable to those observed with cells in medium. The viability of fresh cells in DPBS was significantly reduced (>65%), but the number of cell clumps was rather similar to that observed in medium (Figure 4).

Increasing the storage time increased cell clumping in medium (*r* = 0.635, *P* < 0.001), but not in NS or DPBS (*P* > 0.05) (Figure 4(a)). There was a time-dependent reduction in the viability in all three solutions, but this reduction was most pronounced in NS (*r* = −0.963, *P* < 0.001) and least evident in medium (*r* = −0.726, *P* < 0.001) (Figure 4(b)). A time-dependent increase in the numbers of dead cells was also observed (*r* = 0.668 for the cells in medium, *r* = 0.969 for the cells in normal saline, and *r* = 0.889 for the cells in DPBS, *P* < 0.001) (Figure 4(c)). There was a time-dependent increase in the numbers of apoptotic cells in medium (*r* = 0.784, *P* < 0.001) and a decrease in DPBS (*r* = −0.745, *P* < 0.001), but not in NS (*P* > 0.05) (Figure 4(d)). The percentage of cells or clumps >30 *µ*m in NS was positively correlated with the number of apoptotic cells (*r* = 0.410, *P* < 0.05).

### 3.3. Effect of Freeze-Thawing Procedure on Cell Clumping and Viability

**Figure 5:**
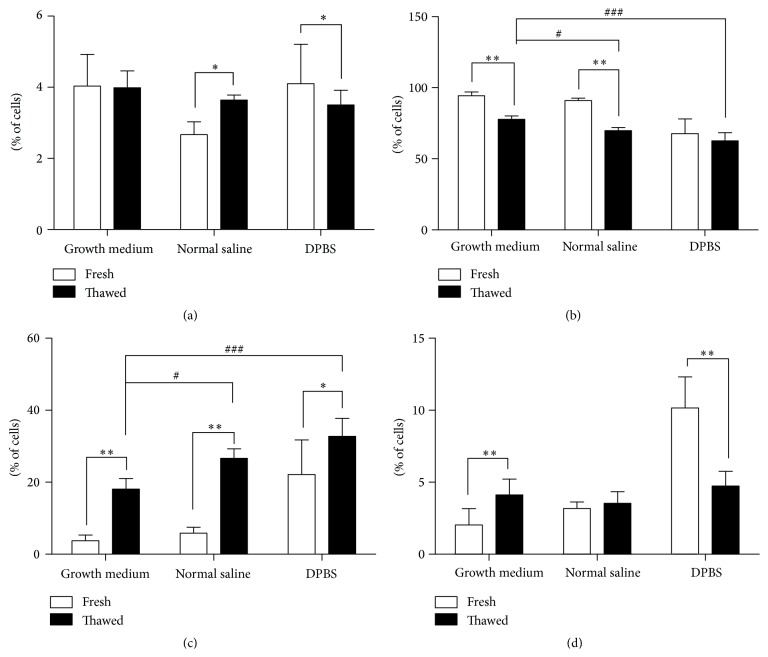
Effect of freeze-thawing procedure on cell clumping and viability of passage 5 rat BMMSCs. (a) Cell clumps or large cells >30 *µ*m; (b) viable cells; (c) dead cells; (d) apoptotic cells. ^*∗*^ *P* < 0.05, ^*∗∗*^ *P* < 0.01; ^#^ *P* < 0.05, ^###^ *P* < 0.001.

In comparison with the fresh cells, a significant increase of cell clumps after thawing was observed for the cells stored in NS (*P* < 0.05), whereas a decrease was observed for cells in DPBS (*P* < 0.05) (Figure 5(a)). There was a significant overall viability reduction of the thawed cells preserved in medium and NS (*P* < 0.01 for both groups), and a trend for those preserved in DPBS suspension (Figure 5(b)). The number of dead cells increased in all conditions (*P* < 0.01 for cells in medium and NS, *P* < 0.05 for cells in DPBS) (Figure 5(c)), and the number of apoptotic cells also increased for the cells in medium (*P* < 0.01) (Figure 5(d)).

The thawed cells stored in medium displayed the highest viability and the lowest percentage of dead cells (*P* < 0.05 compared to the thawed cells in NS, *P* < 0.001 compared to the thawed cells in DPBS) (Figures 5(b) and 5(c)); no significant difference was observed in the percentage of cell clumps or apoptotic cells (Figures 5(a) and 5(d)) across conditions.

## 4. Discussion

Currently there is an urgent need for the standardization of manufacturing, preparation, and transplantation of MSCs [17–19]. Our study provides new insights into MSC preparation by quantifying cell clumping in suspension prior to transplantation and for the first time investigated the effects of several common variables during the cell preparation procedure on both clumping and viability.

Our previous study observed a cell dose-related increase in cerebral embolism evoked after intra-arterial delivery into rats, using a constant volume of rat BMMSCs suspension in DPBS with different cell concentrations [12]. To exclude the possibility of increasing cell clumps with escalating cell concentration in suspension, cells in DPBS with concentrations from 0.2 to 2.0 × 10^6^/mL were assessed in this study. In contrast to our expectations, the data clearly revealed that the degree of cell clumping in suspension did not increase with cell concentration. Therefore, this dose-dependent cerebral embolism could have been associated with cellular interactions in vivo rather than the cell clumps in suspension per se. Increased cell aggregation in vivo, as well as the dose-dependent ability of MSCs to activate blood coagulation due to the expression of tissue factor and collagen type I after transplantation, could account for the cell dose-related safety issues [20, 21]. Moreover, it is also possible that adhesion, cell clumping, and a change in the cell viability took place after delivery through the catheter and needle, but these issues were not evaluated in this study.

Cell viability increased with escalating cell concentrations, indicating that even a 10-fold increase in cell concentration did not exert any direct negative impact on cell viability. However, it also needs to be emphasized that even the highest cell concentration (2 × 10^6^/mL) used in this study is still rather moderate, in comparison to the concentrations of up to 10 × 10^6^/mL of MSCs reported via intra-arterial and 100 × 10^6^/mL via intravenous injection [22, 23].

Phosphate-buffered saline, NS, and cell culture media have been the three most commonly used storage solutions before transplantation of MSCs in animal studies. Our data showed that, after harvesting, the percentage of cell clumps in NS was the lowest with >90% viability. Before cell transplantation, up to several hours may elapse due to transportation and other technical reasons, and this time delay may affect the quality of the cell product. The numbers of cell clumps in medium increased with the storage duration in suspension, probably because the medium contains calcium and magnesium, which are important ions for cell adhesion. The viabilities of the cells in DPBS and NS decreased rapidly, probably due to the lack of nutrients or buffering capacity against exocytosed metabolic end products. Therefore, cells in solutions should be used immediately to ensure that there is neither any increase in cell clumping nor any reduction in cell viability. The number of cell clumps correlated with the number of apoptotic cells for the cells in NS, which might be related to the nucleic acids released by late apoptotic or dead cells.

The freeze-thawing procedure is another controversial factor that might affect cell quality. Cryopreserved cells are good alternatives for cell therapy because of their immediate “off-the-shelf” availability. However, cryopreserved cells have been reported to exert compromised immunomodulatory effects and can induce a more intense immediate blood-mediated inflammatory reaction [24, 25], while some other recent studies have reported opposite findings [26, 27]. In this study, as expected, cryopreserved rat BMMSCs were less viable after thawing than their fresh counterparts with increasing numbers of dead and apoptotic cells, which might affect their therapeutic capabilities. Nevertheless, the changes of cell clumping for the cells in the three types of solutions were different after the freeze-thawing procedure, which could be related with different tendencies towards cell death and apoptosis and the corresponding buffering capacity.

An overestimation of cell viability with the trypan blue method (data not shown) as compared to flow cytometry results was observed in our study, consistent with previous findings from other groups [24, 25]. This discrepancy in viability results is probably due to the presence of apoptotic cells that cannot be detected by trypan blue staining. Since viability results strongly rely on the method used, it is recommended that more sensitive and well-validated viability assays should be utilized in future preclinical and clinical studies of cell therapy.

The flow cytometry-based pulse-width assay provides a clear idea of size distribution and quantification of cell suspension and can also be used for the evaluation of filtering and other techniques to reduce clumps, recommending it as a potential release assay for clinical use. Criteria of clumps ≤5% of nondebris events and ≤150,000 total particles >30 *µ*m have been applied in one study using human bone marrow derived-aldehyde dehydrogenase bright cells, taking into account the number and diameters of human cerebral capillaries [14]. Similar criteria are not available for preclinical studies. However, due to the heterogeneity of preclinical results as well as the discrepancies observed between preclinical and clinical data, it would be advantageous if similar criteria could be devised for individual preclinical studies as this would ensure more consistent results and faster translation into the clinic.

## 5. Conclusions

Cell concentration, storage solution, storage time in suspension, and freeze-thawing procedure can influence cell viability and/or clumping before transplantation. The immediate use of fresh cells in NS seems to contain fewer cell clumps and also with high cell viability, both of which are beneficial for cell transplantation. Quantification of cell clumping can be conducted using the flow cytometry-based pulse-width assay prior to intravenous and particularly intra-arterial cell delivery in order to prevent vascular embolism.

## Abbreviations

MSCs: Mesenchymal stromal cells
BMMSCs: Bone marrow derived mesenchymal stromal cells
FBS: Fetal bovine serum
DPBS: Dulbecco's phosphate-buffered saline
NS: Normal saline
SD: Standard deviation.
